# Single-use negative pressure wound therapy to prevent surgical site complications in high-risk patients undergoing caesarean sections: a real-world study

**DOI:** 10.1093/intqhc/mzad089

**Published:** 2023-10-17

**Authors:** Mendinaro Imcha, Nyan Chin Liew, Arthur McNally, Davor Zibar, Mairead O’Riordan, Aoife Currie, Tim Styche, Jacqui Hughes, Catherine Whittall

**Affiliations:** Obstetrics and Gynaecology, Limerick University Maternity Hospital, Ennis Road, Limerick V94 C566, Republic of Ireland; Obstetrics and Gynaecology, Limerick University Maternity Hospital, Ennis Road, Limerick V94 C566, Republic of Ireland; Obstetrics and Gynaecology, Royal Jubilee Maternity Hospital, 274 Grosvenor Road, Belfast BT12 6BA, UK; Obstetrics and Gynaecology, University College Hospital Galway, Newcastle Road, Galway H91 YR71, Republic of Ireland; Obstetrics and Gynaecology, Cork University Maternity Hospital, Wilton Road, Cork T12 YE02, Republic of Ireland; Obstetrics and Gynaecology, Craigavon Area Hospital, 68 Lurgan Road, Craigavon BT63 5QQ, Northern Ireland; Global HEOR, Smith & Nephew, 101 Hessle Road, Hull HU3 2BN, UK; Global HEOR, Smith & Nephew, 101 Hessle Road, Hull HU3 2BN, UK; Global HEOR, Smith & Nephew, 101 Hessle Road, Hull HU3 2BN, UK

**Keywords:** caesarean section, negative pressure wound therapy, surgical site infection, wound-related complications

## Abstract

Surgical site complications (SSCs), including surgical site infection (SSI), are common following C-sections. Management of the post-operative incision with single-use negative pressure wound therapy (sNPWT) has been shown to reduce the risk of SSC in high-risk individuals. This study explored the outcomes of routine, real-world use of sNPWT in high-risk patients undergoing C-sections.

An observational, retrospective in-service evaluation was conducted across eight obstetric centres in the Republic and Northern Ireland. Patients undergoing C-sections were stratified for their risk of developing SSC using commonly known risk factors, including BMI ≥30, smoking, diabetes, and whether the patients had undergone previous C-sections or had a previous history of wound dehiscence. Those at high-risk were treated with sNPWT post-operatively. Data relating to any SSC that developed post-operatively, for up to 30 days, were captured. Data were compared with original research previously published by Wloch *et al.* (2012).

Of 1111 women considered high-risk, 106 (9.5%) went on to develop SSCs, predominantly superficial SSIs. SSCs were associated with extra visits with their general practitioner (GP), outpatient visits, or inpatient hospital stays in 5.7%, 2.4%, and 1.7% of the entire cohort, representing 59.4%, 25.5%, and 17.9% of the 106 patients with SSC. Patients needed on average 1.8 extra GP visits and 0.7 extra outpatient visits. Patients who needed to be readmitted to hospital had an average length of stay of 4 days. In comparison with a previously published cohort, in which sNPWT was not used, we observed a significant reduction in the incidence of SSCs across BMI groups 18.5–24.9 (*P* = 0.02), 25–29.9 (*P* = 0.003), and ≥35 kg/m^2^ (*P* = 0.04). In those patients who had undergone at least one previous C-section, the rates of complications also reduced (*P* = 0.006).

This analysis provides further justification for using sNPWT to manage surgical incisions in patients considered at high risk of developing post-procedural SSCs, particularly those with a BMI ≥30 or a history of more than one C-section.

## Introduction

Caesarean section (C-section) rates in developed countries have risen [1] due to multiple factors [2]. They are common procedures with worldwide figures increasing annually [2, 3]; globally 1:5 babies are delivered via C-section [1, 4] rising to 1:4 in the UK [5] and 1:3 in Ireland [6].

Although access to C-sections has had a positive impact on newborn and maternal mortality and morbidity [7], post-operative surgical site complications (SSCs), including surgical site infections (SSIs), remain common, affecting around 1:10 women [8, 9]. Risk factors for SSCs can include smoking, diabetes, high body mass index (BMI), and emergency or repeat C-sections [8–11].

Published studies have described the substantial burden SSCs have on local health economies across primary and secondary care [12–14]. This burden includes costs associated with increased length of stay, readmissions, medication, and community care [13, 14]. With post-discharge surveillance not routinely carried out in community settings, the true cost is likely underestimated.

Identifying the risk factors for SSI preoperatively and mitigating the risk of SSCs are important to maximize the likelihood of successful outcomes post-surgery. Despite best-practice surgical guidelines being available [5], the number of SSCs that occur after C-sections remains high [9, 15]. Strategies to address SSI rates have included improved hygiene measures, surgical closure techniques, and timed use of prophylactic antibiotics [16]. Over the past few years, clinical evidence has demonstrated that advanced therapies such as single-use negative pressure wound therapy (sNPWT) may contribute to fewer SSIs when used prophylactically on a variety of closed incisions [12] including C-sections [3, 10], particularly for those patients considered high-risk [17].This approach is consistent with guidance from the National Institute for Health and Care Excellence (NICE) which states that ‘NPWT should be considered an option for closed surgical incisions in people at high -risk of developing SSIs’ [18], and specifically in C-section, to ‘consider NPWT after caesarean birth for women with a BMI≥35 kg/m^2^, to reduce the risk of wound infections’ [5]. The World Health Organization (WHO) also suggests the use of prophylactic NPWT on primary closed surgical incisions in high-risk wounds to prevent SSIs [19].

This study was an observational, retrospective evaluation exploring the real-world use of sNPWT for post-surgical management of higher-risk C-section incisions, with data from previously published cohorts which did not use sNPWT [8], as a comparator. The objective was to explore the impact of sNPWT when used routinely in patients with one or more risk factors for SSC.

## Materials and methods

This observational evaluation of a sNPWT device (PICO™ sNPWT, Smith + Nephew, Hull, UK) was completed across eight hospitals in the Republic of Ireland and Northern Ireland (Belfast, Cork, Craigavon, Drogheda, Galway, Limerick, Portiuncula, and Sligo) between January 2016 and December 2020, and reported in line with the Reporting of studies Conducted using Observational Routinely-collected Data guidelines [20]. For the sites outside the UK, the lead clinician at each centre engaged with their local ethics departments, before confirming with the research team that ethical approval was not required for this type of data collection (it is classified as a service evaluation for data that is captured routinely as standard of care). For the UK Centres, the Health Research Authority deemed this as a service evaluation, not research, and therefore did not require ethical approval. Data Capture Agreements were put in place with each participating centre. These agreements allowed the centres to share anonymized data from patients who had been routinely treated with the NPWT device following their surgery.

**Figure 1:**
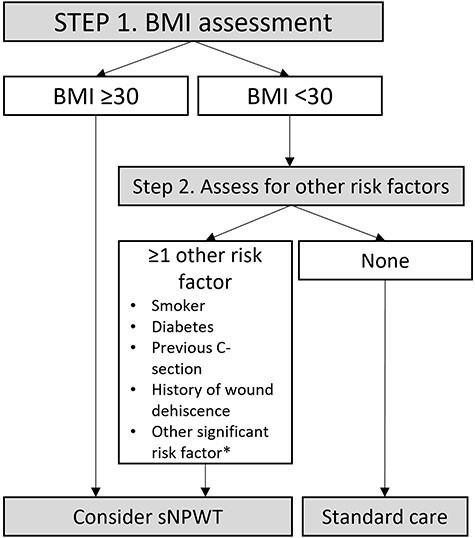
Assessment of risk of wound-related complications. The risk of SSC was determined according to a formalized protocol. *The attending surgeon determined the presence of other significant risk factors not formally itemized on the protocol, according to their clinical judgement.

Clinicians stratified their patient populations for risk of developing SSC, according to a local protocol, developed from national guidelines (NICE) [5, 18]. The protocol is illustrated in Fig. 1. Briefly, patients with a BMI ≥30 kg/m^2^ were considered high-risk for developing SSCs. Patients with BMI <30 kg/m^2^ were assessed for other risk factors, including smoking, diabetes, previous C-section, or wound dehiscence. Patients who did not have these specific risk factors but had other non-itemized risk factors could be identified by the attending surgeon as high-risk, at their discretion and according to clinical judgement. Patients deemed high-risk were considered eligible for treatment with sNPWT. Patients without these risk factors were treated with standard care (traditional post-operative dressings).

Centres observed all elements of standard care for patients, with the only change being the use of the sNPWT device in patients deemed high-risk for SSCs. Where sNPWT was used, clinicians applied the device in theatre immediately after wound closure.

To determine the incidence of SSCs, patients were followed up after 30 days via phone call, to ascertain whether any complications had occurred. If the patient had received antibiotics from their general practitioner (GP) for their incision, this was considered to indicate an SSI.

### Data collection and analysis

Analysis in this report focused on outcomes for the high-risk cohort. Data relating to factors known to influence the likelihood of SSCs were collected retrospectively from patient notes. These factors included BMI, age, smoking, and diabetes. Details of prior or emergency C-sections were also collected.

Data relating to the healing of the surgical incision were captured, including the incidence of post-procedural SSC and the hospital resource impact these complications demanded. SSC was categorized into superficial or deep SSI, as defined by the Centers for Disease Control and Prevention criteria [21], wound dehiscence (in the absence of infection), and ‘other’ such as seroma or haematoma. Data relating to hospital readmission and GP visits in the 30 days following the procedure were also captured.

Data were captured via an online tool (SnapSurveys, London, UK); variables such as age or BMI were collected using category variables. Attempts were made to identify missing data from patient charts. If missing, no values were input or assumed, except for information about hospital readmissions; where readmission was not specified, it was assumed that hospital readmission hadn’t occurred. Data were captured within the SNAP Platform and analysed using SAS 9.4. All data were subject to frequency counts to evaluate population characteristics and rate of complication, with means calculated for resource implications, as appropriate.

### Comparison with previously published data

As this was a non-comparative observational study, the data presented here were compared to the data reported by Wloch *et al.* (2012) [8] in their broader evaluation of SSC risk following C-section. This decision was made prospectively, during study planning. Patients were subdivided into categories that were comparable with those by Wloch *et al.* (2012) [8]. The difference in complication rates was statistically evaluated using Fischer’s exact test. Statistical significance was set at *P* = 0.05.

## Results

Data were collected on 1111 patients who were treated prophylactically with the sNPWT device. Demographic information is shown in Table 1.

**Table 1.** Patient demographics and risk factors.

Patient factor	*N*	%
**Age**		
<30	246	22%
30–39	746	67%
40–49	115	10%
**BMI (kg/m^2^)**		
<18.5	6	1%
18.5–24.9	173	16%
25–29.9 (overweight)	200	18%
30–34.9 (obese)	179	16%
≥35 (extremely obese)	534	49%
**Risk factors**		
Steroids	43	4%
Smoker	94	8%
Diabetes	207	19%
Emergency C-section	539	49%
Previous C-section	430	39%
**Number of risk factors**		
0	7	0.6
1	353	31.8
2	478	43.0
3	217	19.5
4+	56	5.0

BMI, body-mass index.

Two-thirds of patients were aged 30–39 years; 16% of the patients were classed as obese (BMI ≥30) and 49% of patients were considered extremely obese (BMI ≥35). Of the other non-procedural risk factors, diabetes was the most prevalent (19%). Procedural risk factors were frequently observed in this cohort, with 49% of C-sections performed as an emergency and 39% of patients having had at least one prior C-section. Two-thirds of patients (67.5%) had more than one risk factor (Table 1).

Overall, in this cohort, 106 patients (9.5%) developed some form of SSC (Table 2), the majority of which were superficial SSIs (78/106; 73.6%). Second common was wound dehiscence (25/106; 23.6%), followed by deep SSI (8/106; 7.5%). Five patients were reported as having an SSC for ‘other’ reasons, typically haematoma or seroma.

**Table 2.** Incidence of surgical site complication and associated resource burden.

Outcome	Entire cohort (*N* = 1111)	SSC subset (*n* = 106)
Healed, ^a^	1049 (94.4)	NA
Patients with SSC, *n* (%)	106 (9.5)	(100)
Superficial SSI	78 (7.0)	(73.6)
Deep SSI	8 (0.7)	(7.5)
Dehiscence	25 (2.3)	(23.6)
Other^b^	5 (0.5)	(4.7)
**Readmission**		
Proportion needing readmission, *n* (%)	19 (1.7)	(17.9)
Mean number of nights during readmission, *n* (SD)	4.0 (3.0)	NA
Mean additional night per complication	0.85	NA
**Outpatient visits**		
Patients needing outpatient visits, *n* (%)	27 (2.4)	(25.5)
Mean number of visits, *n* (SD)	2.8 (3.0)	NA
Mean number of visits per complication	0.72	NA
**GP visits needed**		
Patients needing GP visit, *n* (%)	63 (5.7)	(59.4)
Mean number of visits, *n* (SD)	1.8 (1.5)	NA
Mean number of visits per complication	1.06	NA

a At time of follow-up (30 days for C-section).

b ‘Other’ risk factors include, for example, seroma or haematoma.

GP, general practitioner; NA, not applicable; SD, standard deviation; SSC, surgical site complication; SSI, surgical site infection.

Data relating to the SSC-related resource burden were captured: 19 patients (1.7%) required readmission for a mean of 4 nights, representing 17.9% of the patients who developed SSCs; 26 patients (2.4%) required outpatient appointments and 63 (5.7%) required consultation with their GP, representing 25.5% and 59.4% of the patients who developed SSC, respectively. Some patients needed multiple visits with an average of 2.8 outpatient appointments and 1.8 GP visits recorded (Table 2).

Results from this study were compared with those previously published in which a broader cohort of patients undergoing C-sections received standard dressings only [8]. They conducted a detailed sub-analysis, investigating the rate of SSC observed using the same risk categories presented in our evaluation (age, diabetes, and BMI) [8]. Although the overall cohort was not directly comparable to our ‘high-risk’ cohort, i.e. it did not include risk factors such as smoking, steroid usage, or previous SSIs, each subset, as stratified according to the presence of risk factors, was directly comparable.

**Figure 2:**
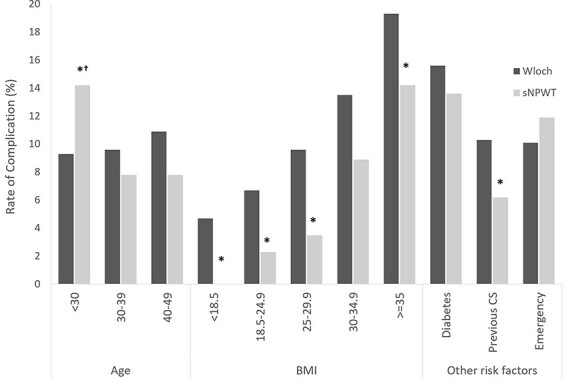
Comparison of complication rate. SSC presented by Wloch *et al.* (2012) in the absence of sNPWT (dark bars) [8] and the present evaluation with sNPWT (lighter bars) are compared by subgroup. The definitions of SSI used by Wloch *et al.* (2012) included clinical signs of infection both in the presence and absence of dehiscence. In our evaluation, clinical signs of infection were not recorded. Here, we assume that in the majority of cases, dehiscence occurred in the context of SSI making data from our evaluation comparable to Wloch *et al.* (2012). *Statistically significant (*P* < 0.05). †In this study, all patients aged <30 had at least one other risk factor which led to their receiving sNPWT. In the Wloch *et al.*’s (2012) study, this age category included all individuals regardless of risk.

BMI, body mass index; SSC, surgical site complication; sNPWT, single-use negative pressure wound therapy.

Comparison of the risk-stratified sub-groups (Fig. 2) demonstrated that in the majority, the rate of SSC in our sNPWT cohort was lower than that reported by Wloch *et al.* (2012) [8], whose patients received standard dressings. Reductions in the rate of SSC following prophylactic sNPWT were seen across all BMI groupings, with significant reductions in categories 18.5–24.9 (6.7% to 2.3%, *P* = 0.02), 25–29.9 (9.6% to 3.5%, *P* = 0.002), and ≥35 (19.3% to 14.2%, *P* = 0.04). A reduction in SSCs in patients with BMI 30–34.9 was also observed although this was not statistically significant (13.5% vs 8.9%; *P* = 0.12). A reduction in SSCs was observed in diabetic patients; however, this finding was not statistically significant (15.6% to 13.6% [8], *P* = 0.58). In patients who had undergone a prior C-section, the rate of SSC with sNPWT was significantly lower than the comparator data [8] (10.3% vs 6.2%, *P* = 0.006); no difference was observed in patients who underwent emergency C-sections (10.1% vs 11.9% in the comparator and sNPWT cohorts, respectively; *P* = 0.17).

## Discussion

### Statement of principle findings

This study reported the results of a real-world evaluation, in which sNPWT was applied to women deemed to be high-risk for developing SSCs. In comparison with a previously published cohort, in which sNPWT was not used, a significant reduction in SSC incidence was observed in several subsets of women at higher risk of developing SSC, including those with high BMI and those who had undergone previous C-sections.

### Interpretation within the context of the wider literature

High BMI has been widely associated with an increased risk of developing SSCs following C-sections [8, 22]. Wloch *et al.*, (2012) [8] reported BMI was a significant factor for SSI, increasing with each successive category. Anderson *et al.* (2013) reported an integrative review specific to C-section; of the 13 relevant papers identified, all agreed obesity increases the risk of SSC [22]. Several studies have demonstrated the ability of sNPWT to reduce the risk of SSC in obese patients [10, 23]. A meta-analysis of randomized controlled studies (RCTs) published by Yu *et al.* (2018) [3] reported a significantly reduced risk of SSC following treatment with sNPWT. Two RCTs compared prophylactic sNPWT with standard post-operative dressings in obese women undergoing C-sections. Both demonstrated a relative risk reduction of SSI in the group assigned to receive sNPWT [10, 23].

In our evaluation, most patients (65%) had a BMI ≥30 as an identified risk factor, reflecting the focus on high-risk individuals. By comparing these results to Wloch *et al.* (2012) [8], we concluded that the adoption of sNPWT may have significantly reduced the incidence of SSC in obese patients. Whilst current recommendations suggest patients with a BMI of >35 undergoing C-sections should have sNPWT for management of the incision to improve outcomes, the trends shown in our evaluation warrant further investigation into preventative strategies for patients with BMI ≥30 kg/m^2^.

The risk of developing an SSI is also increased through certain procedural-related factors [24] Patients with a history of C-sections are documented as being seven times more likely to develop an SSI [9]. Our evaluation reported that the overall SSI rate was significantly lower than that reported by Wloch *et al.* (2012) [8] (6.2% vs 10.3%; *P* = 0.006). It has been suggested that patients who undergo emergency C-sections have an elevated risk of developing an SSI compared to elective surgery, with published increases varying from 1.6 to 3 times greater [10, 11]. In our evaluation, no benefit of sNPWT in comparison to the respective subgroup in Wloch *et al.* (2012) was observed; the reasons for this are unknown. For emergency C-sections, there are multiple reasons around urgent care situations why the two groups may not have been comparable; however, that information is not available for us to explore because it wasn’t captured in any greater depth. As such, no robust conclusion can be drawn from our study around this cohort of patients.

In this evaluation, patients in the <30 age group had a significantly higher incidence rate of complications compared to the Wloch *et al.*’s (2012) data (9.3% vs 5.6%, *P* < 0.02). Importantly, all patients aged <30 in our evaluation had at least one other risk factor which led to their receiving sNPWT. In the Wloch *et al.*’s (2012) study, this age category included all individuals regardless of risk and so we are not able to draw a similar comparison with this dataset.

Some of the consequences of SSCs include increased care requirements and associated costs [13]. Whilst the majority of SSIs are treated in the community, hospital readmissions do occur in this cohort, resulting in increased costs and a direct impact on service delivery [14]. The median additional length of stay (LOS) attributable to SSI following C-section has previously been reported to be 4 days, with an associated cost of £3700 per patient [25]. The objective of reducing hospital admissions should not be in isolation, as the burden placed on community services can also be significant, resulting in increased visits from community midwives and GPs in addition to the cost of antibiotics [14]. Our evaluation showed that around 60% of patients who developed complications needed an average of 1.8 visits with their GP as a direct consequence. In the Wloch study [8], of the 394 patients who developed a wound complication, only 23 (0.6%) were readmitted to hospital; in our evaluation, we observed a readmission rate of 19/106 (17.9%). This difference in readmission rates could be due to differences in the overall patient group as, unlike the Wloch *et al.*’s (2012) [8] study, our evaluation only included high-risk patients, the majority of whom had multiple risk factors. There were 76 additional nights due to readmission observed, an average of 4 nights per patient (*n* = 19); 26% of the readmissions were due to deep SSIs, and 15.8% were due to the patient having a dehisced wound following a superficial SSI.

### Strengths and limitations

This study had several strengths and limitations; the evaluation reflected real-world clinical practice and as such, has wider applicability to everyday practice than some rigorously designed studies. This approach has been successfully adopted in other indications [26]. The study design, however, has several limitations. Firstly, the subgroup data from the Wloch *et al.*’s (2012) [8] study were used as a benchmark for the incidence of SSC without sNPWT. Although there were fundamental differences between the overall populations described, (Wloch *et al.* (2012) [8] was conducted on an all-comer population and our evaluation was conducted on a high-risk population, we controlled for this by limiting the analysis to comparing well-defined risk-stratified subsets that were consistent between both papers. Another difference was how SSCs were defined in each study; Wloch *et al.* (2012) included dehiscence only in the context of SSI (i.e. in the presence of individual clinical signs of infection). In our evaluation, dehiscence was identified as an SSC regardless of whether signs of infection were present or absent. Our comparison with Wloch *et al.* (2012) [8] assumed that all dehiscence events occurred in the context of SSI and were therefore directly comparable to the Wloch data set. However, dehiscence events that occurred in the absence of infection may have been included in our dataset; had these been removed from the analysis, to enable a like-for-like comparison, the impact of our evaluation’s results may have been further enhanced [8]. It is also assumed that the majority of procedures within this evaluation were conducted by a consultant; however, the limitation of this dataset is that the seniority of the Clinician was not captured. It was demonstrated by Wloch *et al.* (2012) [8] that there was a decrease in subsequent SSIs if a consultant conducted the operation compared to other grades; we are not able to draw a similar comparison with this dataset. Further, for the cohorts of patients who had undergone a previous C-section, it was recorded within this evaluation whether the patient had undergone one or more previous C-sections; however, the exact number was not recorded; this is a limitation that could be explored further in future research. We cannot rule other out other potential sources of heterogeneity between our patient cohort and those presented by Wloch *et al.* (2012) [8]. However, we are encouraged by the fact that statistically significant reductions were observed in the risk-stratified sub-groups in a manner consistent with other higher-level studies. Secondly, chart reviews, as described in this evaluation, can be subject to unmeasured confounding factors and can be affected by incomplete data sets.

### Implications for policy, practice, and research

This analysis demonstrates a clear clinical benefit of the use of sNPWT in women undergoing C-sections who are considered high-risk for developing SSCs. Before the implementation of the protocol described, the use of sNPWT was *ad-hoc* and at the discretion of clinicians. The corollary of this is the requirement for improved clinical protocols and guidelines on the use of sNPWT, available at local, national, and international levels. Our analysis was part of a quality improvement programme, designed to implement a local clinical protocol in several institutions across Ireland. Implementation of the protocol maximized the benefits of sNPWT by formalizing the clinical scenarios in which sNPWT should be used for greatest benefit across the population.

## Conclusions

This analysis strengthens the justification for using sNPWT to manage incisions in patients considered high-risk for developing SSCs; this supports existing recommendations [5]. This analysis also suggests that it may be justifiable to extend the use of sNPWT to other patient subgroups, including individuals who have had a previous C-section with no other risk factors. The results of this evaluation are encouraging, and the significance of the findings and the significant existing evidence should give some assurance regarding the validity of the study. Healthcare establishments are encouraged to review local post-operative wound care protocols to ensure that positive clinical and health-economic outcomes are achieved by mitigating the risk of developing SSC following C-section in patients with identified risk factors.

## Data sharing statement

The data underlying this article will be shared on reasonable request to the corresponding author.
